# Human Epididymis Protein 4 Predicted Concurrent Intermediate-high-risk Endometrial Cancer and Eligibility of Fertility-sparing Treatment for Patients Diagnosed with Endometrial Atypical Hyperplasia Before Surgery

**DOI:** 10.7150/ijms.115170

**Published:** 2025-07-11

**Authors:** Yaochen Lou, Weirong Ma, Feng Jiang, Jun Guan

**Affiliations:** 1 Department of Gynecology, Obstetrics & Gynecology Hospital of Fudan University, Shanghai Key Lab of Reproduction and Development, Shanghai Key Lab of Female Reproductive Endocrine Related Diseases, 200433, Shanghai, China.; 2 Department of Gynecology, Affiliated Hospital of Nanjing University of Chinese Medicine, Jiangsu Province Hospital of Chinese Medicine, 210029, Nanjing, China.; 3 Department of Neonatology, Obstetrics & Gynecology Hospital of Fudan University, Shanghai Key Lab of Reproduction and Development, Shanghai Key Lab of Female Reproductive Endocrine Related Diseases, 200433, Shanghai, China.

**Keywords:** human epididymis protein 4, endometrial cancer, endometrial atypical hyperplasia, intermediate-high-risk, fertility-sparing treatment

## Abstract

**Objective:** To investigate whether serum human epididymis protein 4 (HE4) could identify concurrent intermediate-high-risk endometrial cancer (EC) in patients diagnosed with endometrial atypical hyperplasia before definitive surgery (preoperative-EAH).

**Methods:** This retrospective study analyzed preoperative-EAH patients who underwent hysterectomy at a tertiary hospital between January 2016 and December 2022.

**Results:** Among 715 preoperative-EAH patients, 26.2% (187/715) were diagnosed with concurrent EC postoperatively, with 6.0% (43/715) identified as having concurrent intermediate-high-risk EC. Serum HE4 and postmenopausal status were revealed as independent predictors of concurrent EC. Receiver operator characteristic analyses determined optimal HE4 cut-off values of 43.50 pmol/L for predicting concurrent EC, 53.15 pmol/L for intermediate-high-risk EC, and 43.80 pmol/L for identifying non-candidates for fertility-sparing treatment. Multivariate analyses confirmed HE4 and postmenopausal status as key independent predictors of intermediate-high-risk EC, leading to the development of a nomogram model. It demonstrated a bootstrap-corrected C-index of 0.819 (95% confidence interval [CI] = 0.74-0.90). The calibration and decision curves highlighted its consistency and clinical utility. According to the nomogram, 41.4% (24/58) of high-score patients had concurrent intermediate-high-risk EC, compared with only 2.9% (19/657) in the low-score group (*P* < 0.001). HE4 also significantly predicted the non-candidates for fertility-preserving therapy in young preoperative-EAH women (odds ratio [OR] = 5.21, 95% CI = 2.10-12.89, *P* < 0.001).

**Conclusion:** Serum HE4 was a promising predictor of concurrent intermediate-high-risk EC and suitability for fertility-sparing treatment for preoperative-EAH patients. Incorporating HE4 and menopausal status into the nomogram model significantly enhanced the risk stratification for intermediate-high-risk EC and might assist clinical decision-making.

## Introduction

Endometrial atypical hyperplasia (EAH) is the precursor lesion of endometrial cancer (EC) 1. For women initially diagnosed with EAH through endometrial biopsy (preoperative-EAH), the standard treatment is a total hysterectomy combined with bilateral salpingectomy if fertility preservation is not required 2. However, almost 40% of preoperative-EAH patients were diagnosed with EC after definitive surgery, and 10-13% of them were high-risk patients 3, 4 who may require a re-staging surgery to evaluate lymph node metastasis to guide adjuvant therapy 5. Although adding sentinel lymph node (SLN) mapping into initial surgery has been suggested to improve the detection of advanced EC 6, 7, this approach may not be generally applied for all preoperative-EAH patients because of potential overtreatment and increased costs. In this context, high-risk patients subsequently underwent systematic lymphadenectomy that associated with increased complications, such as lymphedema and chylous leakage, leading to prolonged recovery and delayed treatment. Additionally, young women with preoperative-EAH often preferred fertility-preserving therapy rather than definitive surgery, leading to serious delay of operation timing and poor prognosis for high-risk patients. Thus, it is crucial to adequately identify potential high-risk patients with preoperative-EAH.

Currently, limited studies have investigated predictors for concurrent EC risk across subtypes, such as one reported cancer antigen 125 (CA125) levels ≥ 35 U/mL correlated with undiagnosed intermediate-high-risk EC in 130 preoperative-EAH patients 8. However, reliable methods for stratifying patients with coexisted higher-risk EC and applicable predictive models for clinical use are lacking. Recent studies have highlighted human epididymis protein 4 (HE4) as a promising serum biomarker for EC prediction due to its cost-effectiveness and accessibility 9-11. However, its effectiveness in identifying preoperative-EAH patients with coexisted higher-risk EC remains uncertain.

This study aimed to investigate whether serum HE4 might 1) be correlated with aggressive clinicopathological characteristics of preoperative-EAH patients, 2) have a predictive effect on concurrent intermediate-high-risk EC (excluding low-risk EC) and might help to establish a nomogram model for clinical use; and 3) help to identify young preoperative-EAH who might not be considered as candidates for fertility-sparing therapy.

## Materials and Methods

### Study population

This retrospective study enrolled preoperative-EAH patients who underwent definitive surgery at the Obstetrics and Gynecology Hospital of Fudan University from January 2016 to December 2022. Eligible patients were those who met the following criteria (Figure S1): 1) were diagnosed with EAH through Pipelle biopsy or dilation and curettage (D&C) with or without hysteroscopy (HSC); (2) underwent definitive surgery within three months after endometrial biopsy; (3) received no fertility-sparing treatment within six months before hysterectomy; (4) had no other malignant tumors; (5) had available clinicopathological data and serum HE4 levels that tested within one month before definitive surgery. The study was approved by the Ethics Committees in this hospital (protocol code: 2021-185), and all the participants consented to their medical information and laboratory data being used for research purposes.

### Diagnosis

Pathological diagnoses were performed by two experienced gynecological pathologists at the hospital according to the World Health Organization (WHO) pathological classification of tumors of the uterine corpus (2020) 12. The diagnosis of EC was staged using the staging system of the International Federation of Gynecology and Obstetrics (FIGO) 2009 guidelines 13, 14.

The intermediate-high-risk EC was defined as all the EC subgroups excluding low-risk EC. According to NCCN guidelines (version 3.2024), the low-risk EC was defined as 1) endometrioid endometrial cancer, stage IA (FIGO2009), grade 1-2, without lymph-vascular space invasion, age < 60 years; or 2) endometrioid endometrial cancer, stage IA (FIGO2009), grade 3, without lymph-vascular space invasion, age < 60 years, without myometrial invasion 5. Correspondingly, all other EC cases except low-risk EC cases were defined as “intermediate-high-risk EC” in this study, based on the NCCN guidelines (version 3.2024) 5.

In all preoperative-EAH patients, the potential candidates for fertility-sparing treatment were defined as: 1) young preoperative-EAH patients with age ≤ 45 years and premenopausal status; 2) had final pathological results strictly met the following criteria: endometrioid histology, grade 1, with no myometrial invasion, no cervical interstitial invasion, no adnexal involvement, no lymph node or distant metastasis. Correspondingly, all other patients who did not meet the above criteria were defined as “non-candidates”, who were consider being unsuitable for fertility preservation in this study.

### Data collection and evaluation

Clinical data and laboratory data were collected. Body mass index (BMI) was calculated as weight (kg) / height (m^2^), with obesity defined as BMI ≥ 28 kg/m^2^ based on the Chinese population standard 15. Metabolic parameters were assessed using laboratory data. The serum creatinine (Scr) was analyzed with a Hitachi 7600 automatic chemistry analyzer (Hitachi Diagnostics Ltd.), and the estimated glomerular filtration rate (eGFR) index was derived using a formula: eGFR (ml/min/1.73m^2^) = 175 × [Scr (mg/dl) ^-1.234^] × [age (years) ^-0.179^] × 0.79 according to Chinese eGFR Investigation Collaboration 16. And eGFR < 90 ml/min/1.73m^2^ was considered as impaired renal function 17. The levels of HE4 and CA125 serum markers were analyzed by Roche COBAS e 601 electrochemiluminescence analyzer (Roche Diagnostics Ltd.). Additionally, pathological reports of endometrial biopsy and hysterectomy were collected.

### Sample size calculation

In our research, a total of 715 participants were included, among whom 43 cases (6.0%) were classified as high-intermediate risk. Aiming to develop a logistic regression model to identify high-intermediate risk EC, this study incorporated four independent variables in the multivariate logistic analysis. According to the recommendation by Peduzzi et al. 18, the minimum requirement for model stability—namely, at least 10 events per variable (EPV)—was satisfied. Further, for a more precise calculation of the sample size, formula-based calculations were applied. Based on an assumed Nagelkerke R² of 0.1 to 0.15 and a significance level (α) of 0.05, the estimated statistical power of the current sample size was approximately 0.76 to 0.85, which was close to or met the conventional threshold of 0.8 for adequate power 19, 20.

### Statistical analysis

The Shapiro-Wilk and Kolmogorov-Smirnov tests were employed to assess the normality of continuous variables. Levene's test examined variance homogeneity. For normally distributed data, the results were presented as 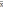
±s, otherwise, medians and interquartile ranges were reported. Two-group comparisons utilized independent t-tests for normally distributed data or Mann-Whitney U tests when data were not normally distributed. Categorical data were presented as frequencies and percentages, and differences between groups were assessed with Chi-squared or Fisher's exact tests. Spearman correlation analysis evaluated the associations between serum HE4 levels and clinical as well as pathological factors in preoperative-EAH patients.

Receiver operating characteristic (ROC) curve analysis determined optimal serum HE4 cut-off values to predict concurrent EC, intermediate-high-risk EC, or patients who were non-candidates for fertility-sparing treatment. This analysis also assessed sensitivity, specificity, and predictive values. The Youden index was calculated as: Youden index = sensitivity + specificity - 1.

Univariate and multivariate logistic regression analyses were used to identify predictors of concurrent EC, intermediate-high-risk EC, and non-candidates for fertility-sparing treatment. Under the univariate analysis, variables that had a *P*-value < 0.05 or clinical significance were included in the multivariate analysis, providing adjusted OR and 95% CIs.

A nomogram was developed to predict intermediate-high-risk EC based on key factors identified from the multivariate analysis. The Akaike information criterion (AIC) and Bayesian information criterion (BIC) were used to evaluate model fit, while the bootstrap-corrected concordance index (C-index) measured predictive accuracy. Calibration and decision curve analysis (DCA) assessed model consistency and clinical benefit. Internal validation employed a 1000-sample bootstrap method. The nomogram provided each preoperative-EAH patient with an individual risk score, which was used to categorize patients into low- and high-risk groups.

Statistical analyses were conducted using SPSS 25.0 (Chicago, USA) and R software (version 4.4.1). Statistical significance was defined as a two-tailed *P* < 0.05.

## Results

A total of 1237 preoperative-EAH patients underwent definitive surgery. Among them, 715 women met the eligibility criteria and were included (Figure S1). Approximately 26.2% (187/715) of the patients were eventually diagnosed with EC (final-EC), of whom 98.4% were classified as endometrioid EC.

The clinicopathological characteristics of eligible patients were presented in Table 1. Compared with patients diagnosed with EAH by definitive surgery (final-EAH), final-EC patients were older, more likely to be postmenopausal, with higher rates of diabetes, hypertension, and thicker presurgical endometrium. Serum HE4 level was also significantly higher in the final-EC group than in final-EAH group (median 52.70 pmol/L vs. 45.94 pmol/L, *P* < 0.001).

Table 2 presented the correlations between serum levels of HE4 and clinicopathological characteristics. Among preoperative-EAH patients, elevated HE4 levels were significantly associated with older age, postmenopausal status, hypertension, higher CA125 levels, and increased endometrial thickness (Table 2). Also, in the final-EC group, higher HE4 levels were significantly linked to malignant characteristics such as deeper myometrial invasion, MELF (microcystic, elongated, and fragmented) positivity, larger tumor size, and diagnosis of intermediate-high-risk EC (Table 2).

We further investigated the predictive capacity of serum HE4 levels for concurrent EC, concurrent intermediate-high-risk EC, and non-candidates for fertility-sparing treatment among preoperative-EAH patients. The cut-off of HE4 was 43.50 pmol/L to predict concurrent EC, which was consistent with our previous study 8 (area under the curve [AUC] = 0.683, 95% CI = 0.64-0.73, *P* < 0.001; Figure S2A). Remarkably, for intermediate-high-risk EC, the predictive capability of HE4 levels was stronger (AUC = 0.774, 95% CI = 0.70-0.85, *P* < 0.001; Figure S2B). At the maximum Youden index of 0.443, the optimal cut-off value of HE4 was 53.15 pmol/L, with a good sensitivity of 0.721 and specificity of 0.722. Meanwhile, when detecting potential non-candidates for fertility-sparing treatment, the cut-off value of HE4 was down to 43.80 pmol/L (AUC = 0.682, 95% CI = 0.59-0.77, *P* < 0.001; Figure S2C). It achieved a high sensitivity of 0.842 but with a modest specificity of 0.494 at the maximum Youden index of 0.336.

**Figure 1:**
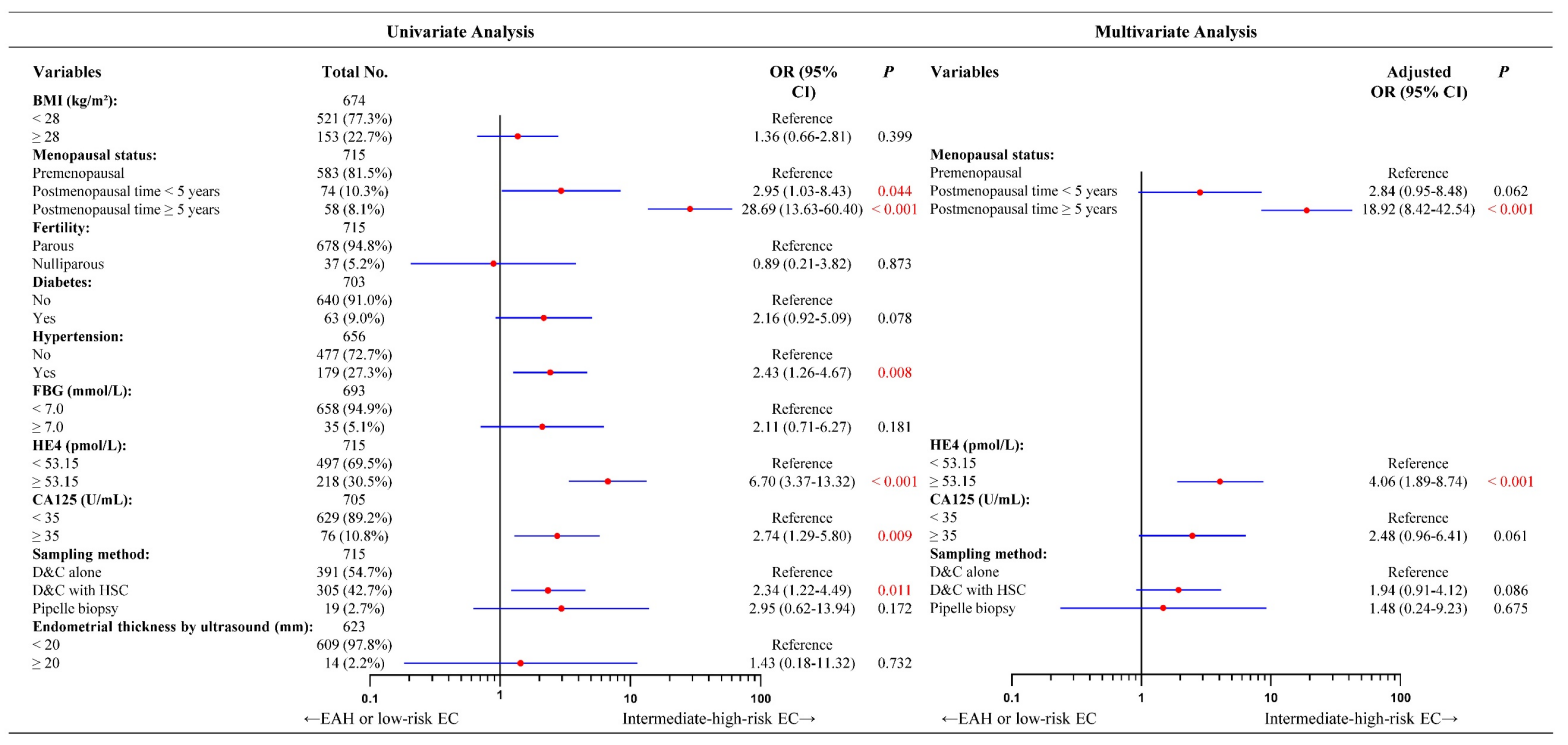
Univariate and multivariate analyses of factors predicting concurrent intermediate-high-risk EC for 715 preoperative-EAH patients based on 2024 NCCN guidelines. Notes: Missing data included 41 cases for BMI, 12 for diabetes, 59 for hypertension, 22 for FBG, 10 for CA125, and 92 for endometrial thickness by ultrasound. The categorical variable of serum HE4 levels had a cut-off as 53.15 pmol/L. Given the relatively high proportion of missing data for hypertension (8.3%, 59/715), and considering the EPV principle and model robustness, hypertension was excluded from the final model. In multivariate logistic analysis, OR adjusted for age, menopausal status, HE4 value, and CA125 value. Significant *P* value < 0.05. Low-risk EC was defined as: (a) endometrioid endometrial cancer, stage IA (FIGO2009), grade 1-2, without lymph-vascular space invasion, age < 60 years; (b) endometrioid endometrial cancer, stage IA (FIGO2009), grade 3, without lymph-vascular space invasion, age < 60 years, without myometrial invasion. All other EC cases except for low-risk EC were defined as intermediate-high-risk EC, based on the NCCN guidelines (version 3.2024). Abbreviations: EAH, endometrial atypical hyperplasia; EC, endometrial cancer; BMI, body mass index; FBG, fasting blood glucose; HE4, human epididymis protein 4; CA125, cancer antigen 125; OR, odds ratio; CI, confidence interval; D&C, dilatation and curettage; HSC, hysteroscopy.

Consistent with our previous findings, HE4 and postmenopausal status were independent predictors of concurrent EC (Figure S3). For concurrent intermediate-high-risk EC, the univariate analysis presented five significantly predictive factors such as elevated HE4 levels, long postmenopausal time, hypertension, higher CA125 levels, and sampling methods (Figure 1). Incorporating the five factors into a multivariate logistic regression model, hypertension did not reach statistical significance (Figure S4). Given its relatively high proportion of missing data (8.3%, 59/715), and considering the EPV principle and model robustness, hypertension was excluded from the final model. The remaining four factors were incorporated into multivariate logistic regression analysis. However, only high HE4 levels and long postmenopausal time remained significant in multivariate analysis. As shown in Figure 1, preoperative-EAH patients with HE4 levels ≥ 53.15 pmol/L had a fourfold higher likelihood of being finally diagnosed with intermediate-high-risk EC than those with lower HE4 levels (OR = 4.06, 95% CI = 1.89-8.74, *P* < 0.001). Similarly, preoperative-EAH women who had < 5 postmenopausal years might be nearly 3 times more likely to develop the intermediate-high-risk EC than those with premenopausal status (OR = 2.84, 95% CI = 0.95-8.48,* P* = 0.062). When postmenopausal years extended to ≥ 5 years, this risk rose to nearly 19 times higher (OR = 18.92, 95% CI = 8.42-42.54, *P* < 0.001).

**Figure 2:**
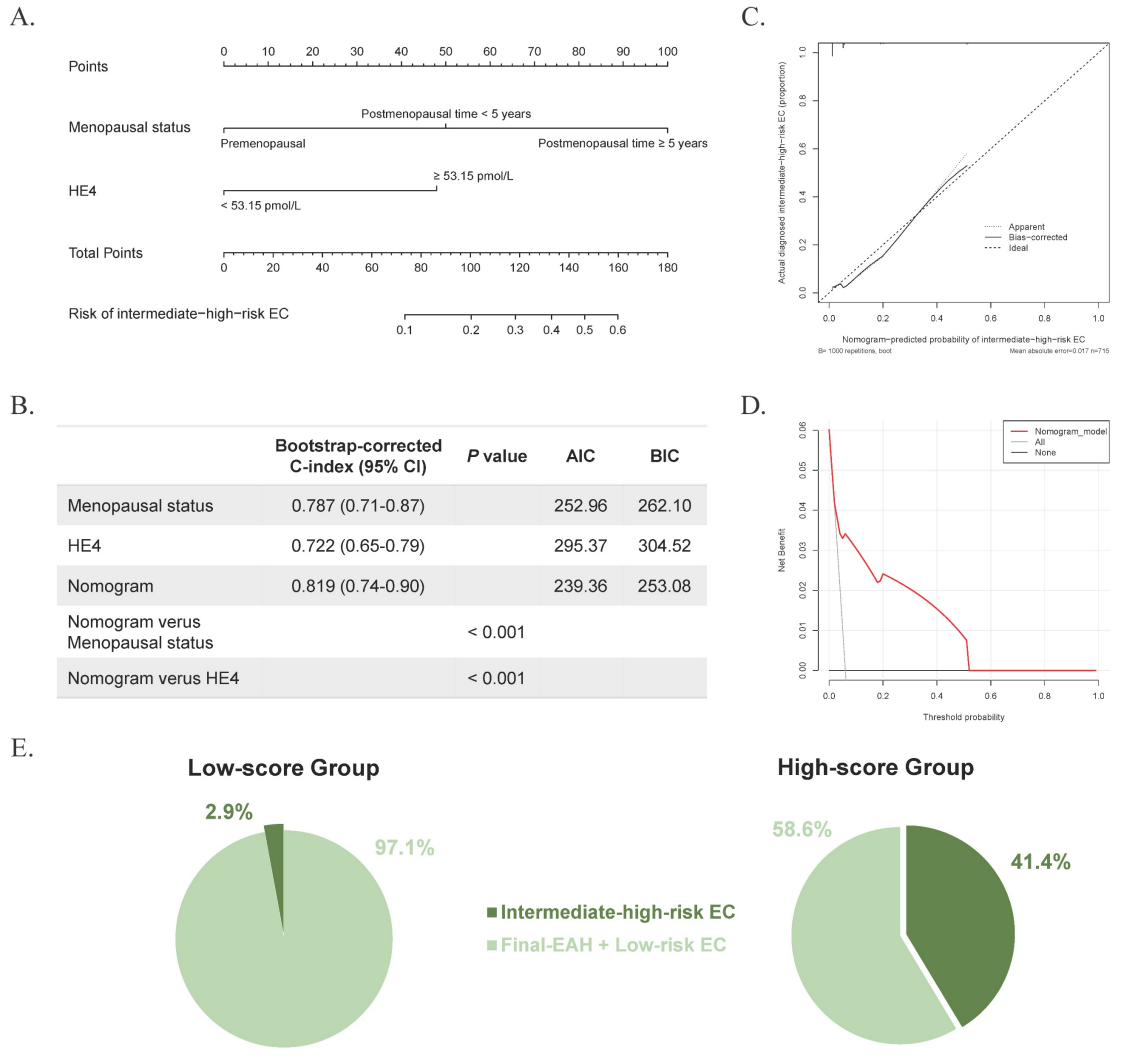
The developed nomogram, and the examination of discriminative ability and net benefit. (A) The developed nomogram based on age, menopausal status, and HE4 for predicting concurrent intermediate-high-risk EC in preoperative-EAH patients, according to 2024 NCCN guidelines. (B) The AIC, BIC, and bootstrap-corrected C-index of prognostic factors and nomogram. (C) The calibration curve of the nomogram prediction. (D) The decision curve analysis of the nomogram prediction. (E) The distribution of concurrent intermediate-high-risk EC in low-score and high-score groups based on the total points of nomogram, according to 2024 NCCN guidelines. Notes: Significant *P* value < 0.05. Low-score, total points < 100; high-score, total points ≥ 100. Low-risk EC was defined as: (a) endometrioid endometrial cancer, stage IA (FIGO2009), grade 1-2, without lymph-vascular space invasion, age < 60 years; (b) endometrioid endometrial cancer, stage IA (FIGO2009), grade 3, without lymph-vascular space invasion, age < 60 years, without myometrial invasion. All other EC cases except for low-risk EC were defined as intermediate-high-risk EC, based on the NCCN guidelines (version 3.2024). Abbreviations: EAH, endometrial atypical hyperplasia; EC, endometrial cancer; HE4, human epididymis protein 4; C-index, concordance index; CI, confidence interval; AIC, Akaike Information Criterion; BIC, Bayesian Information Criterion.

Based on these predictors, a nomogram was established for predicting intermediate-high-risk EC (Figure 2A). The C-index was 0.818 and remained at 0.819 (95% CI = 0.74-0.90) with bootstrap-corrected validation, outperforming each individual predictive factor (Figure 2B). Also, the nomogram presented a satisfied goodness-of-fit, with AIC and BIC values (239.36 and 253.08, respectively) lower than those of individual predictive markers (Figure 2B). Meanwhile, the calibration curve showed good agreement between prediction and observation for the probability of intermediate-high-risk EC (Figure 2C). And the DCA revealed a net benefit to predict intermediate-high-risk EC with a threshold probability range of 2%-51% (Figure 2D). According to the nomogram, all 715 preoperative-EAH patients were divided into low-score (total points < 100) and high-score (total points ≥ 100) subgroups. Notably, the proportion of high-score patients who were finally diagnosed with intermediate-high-risk EC exceeded two-fifths (41.4%, 24/58), whereas among low-score patients it was less than 3% (2.9%, 19/657,* P* < 0.001, Figure 2E), indicating the excellent discrimination of the predictive model. The sensitivity, specificity, positive predictive value, and negative predictive value were 0.558, 0.949, 0.414, and 0.971, respectively.

**Figure 3:**
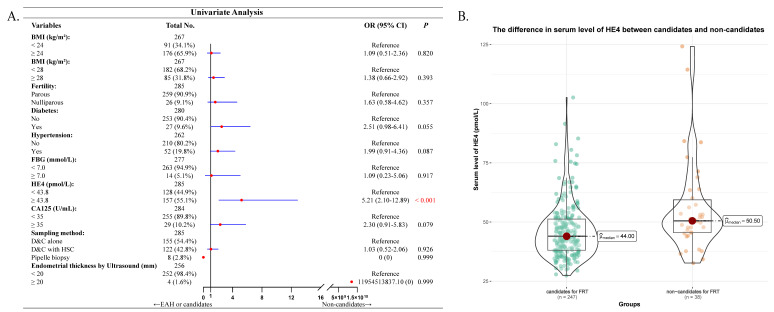
Correlation between serum HE4 and non-candidates for fertility-preservation therapy (FPT) in 285 young preoperative-EAH patients. (A) Univariate regression model of factors including serum HE4 that predicted non-candidates for FPT. (B) The difference in serum level of HE4 between candidates and non-candidates for FPT. Notes: In (A), Missing data included 18 cases for BMI, 5 for diabetes, 23 for hypertension, 8 for FBG, 1 for CA125, and 29 for endometrial thickness by ultrasound. The categorical variable of serum HE4 levels had a cut-off as 43.8 pmol/L. Significant *P* value < 0.05. Abbreviations: EAH, endometrial atypical hyperplasia; EC, endometrial cancer; BMI, body mass index; FBG, fasting blood glucose; HE4, human epididymis protein 4; CA125, cancer antigen 125; OR, odds ratio; CI, confidence interval; FPT, fertility-preserving therapy; D&C, dilatation and curettage; HSC, hysteroscopy.

At last, we analyzed the factors that might predict the potential non-candidates for fertility-preserving treatment in 285 young preoperative-EAH women. Serum HE4 was significantly higher in non-candidates compared to candidates (median 50.50 pmol/L vs. 44.00 pmol/L, *P* < 0.001, Figure 3B). However, only elevated serum HE4 succeeded the predictive significance in univariate analysis (OR = 5.21, 95% CI = 2.10-12.89, *P* < 0.001, Figure 3A). Multivariate analysis was not further performed.

## Discussion

In this study, we found that higher levels of serum HE4 were significantly associated with older age, postmenopausal status, hypertension, higher serum CA125, and increased endometrial thickness in preoperative-EAH patients. Additionally, elevated HE4 were linked to more aggressive pathological features in patients with concurrent EC, including deeper myometrial invasion, MELF positivity, larger tumor size, and the diagnosis of intermediate-high-risk EC. Notably, serum HE4 was found to independently predict concurrent intermediate-high-risk EC, and this predictive ability was significantly improved after incorporating menopausal status into a nomogram model. This model was good for clinical practice, as it helped stratify preoperative-EAH women who were at a higher risk of concurrent intermediate-high-risk EC (41.4% vs 2.9% in the high-score and low-score groups). Furthermore, among young women with preoperative-EAH, higher serum levels of HE4 were significantly correlated with a poor outcome that was ineligible for fertility-sparing treatment. In addition, we revealed that the cut-offs of HE4 levels were varied across populations, showing that the highest cut-off value of HE4 was most effective for predicting concurrent intermediate-high-risk EC, compared to predictions of concurrent EC or non-candidates for fertility preservation.

The clinical significance of predicting concurrent EC in preoperative-EAH patients has been underlined in our study. Those findings were consistent with our previous research, although the sample size was 2-folder larger in this study 21. Previously, our nomogram model incorporated serum HE4 levels of 43.5 pmol/L, menopausal status, and BMI to stratify patients into low- and high-risk groups 21. Notably, nearly half of the patients in the high-risk group were found to have concurrent EC with all subtypes 21. However, women with low-risk EC can receive observation after incomplete staging, whereas patients with intermediate-higher-risk EC usually require a re-staging surgery to assess lymph node metastasis. In this study, we improved the risk stratification for concurrent intermediate-high-risk EC. After developing a nomogram including serum level of HE4 ≥ 53.15 pmol/L and postmenopausal status, we discovered that 41.4% of patients in the high-score group were eventually diagnosed with intermediate-high-risk EC, compared to only 2.9% of the low-score group. These findings might prompt a recommendation for systemic imaging evaluations before definitive surgery and for SLN mapping to evaluate lymph node metastasis, prevent second procedures, and reduce surgical injury. Furthermore, according to the nomogram, better counselling could be conducted for patients, providing informed consent on the risk of pathological upgrading and the possibility of close follow-up. Collectively, our results might optimize the risk stratification of concurrent intermediate-high-risk EC and assist clinical decision-making for preoperative-EAH women.

This study established the first model to predict intermediate-high-risk EC in preoperative-EAH populations with internal validation. In a previous retrospective study, we tried to identify risk factors for intermediate-high-risk EC in preoperative-EAH patients 8. It revealed that serum CA125 ≥ 35 U/mL might be a predictive factor 8. However, consensus on risk factors has yet to be reached, and no predictive model has been established due to the lack of centralized pathological review. These risk markers have not demonstrated practical value in clinical settings. HE4 was a glycoprotein expressed in various tissues, and it has shown a significant correlation with EC 22-26. However, research on the relationship between HE4 and EAH is relatively lacking. Two retrospective studies explored incorporating HE4 in preoperative models for endometrial intraepithelial neoplasia or EAH patients 21, 27. One reported that HE4 levels ≥ 43.50 pmol/L, BMI ≥ 28 kg/m², and postmenopausal status might predict concurrent EC 21. The other one found that increased endometrial thickness (≥ 15 mm), menopause, hypertension, HE4 levels, and endometrial blood were significantly associated with upstaging 27. In both studies, HE4 emerged as an independent predictive factor, highlighting its predictive value. However, the association between HE4 and intermediate-high-risk EC lack analyses. Our current study identified HE4 as an independent predictor of concurrent intermediate-high-risk EC, demonstrating superior efficacy compared to predicting all EC subtypes (for concurrent intermediate-high-risk EC: OR = 4.06, 95% CI = 1.89-8.74, *P* < 0.001; for concurrent EC: OR = 2.61, 95% CI = 1.68-4.08, *P* < 0.001). This led to a more precise predictive model for individual clinical decision-making.

This study identified correlations between high HE4 levels and both malignant clinicopathological features and poor outcomes in preoperative-EAH patients. Previous studies have explored that the increased serum HE4 levels were associated with higher disease severity and progestin-treatment failure 28, 29. Some studies have incorporated HE4 into predictive models for concurrent EC in preoperative-EAH patients 21, 27. However, its value as a biomarker in these populations is still not well investigated. We discovered that elevated serum HE4 levels were significantly associated with older age, postmenopausal status, hypertension, elevated CA125 levels, and thicker endometrium in preoperative-EAH patients. In final-EC patients, increased HE4 levels were related to more severe pathological features including deeper myometrial invasion, MELF positivity, larger tumor size, and intermediate-high-risk EC. These findings aligned with previous studies on EC patients 9, 30, suggesting that HE4 might predict poor outcomes in preoperative-EAH patients.

Remarkably, HE4 presented a promising predictive value in accurately identifying non-candidates for fertility preservation among preoperative-EAH patients, with a defined cut-off value of 43.80 pmol/L. However, the predictive model was not expanded due to the absence of other predictors. Prior research has linked HE4 levels with fertility preservation outcomes and response to treatment 28, 29. Higher baseline serum HE4 levels independently predicted resistance to treatment with the levonorgestrel-releasing intrauterine system in early-stage EC (EEC) and EAH patients 29. Each 1 pmol/L increase in HE4 reduced the treatment response likelihood by 3% 28. According to our findings, if preoperative-EAH women with high HE4 levels demand fertility-sparing treatment, they should be informed of the potentially high risk of treatment failure and concurrent cancer. Closer monitoring and timely intervention should be also suggested for these fertility-sparing patients. However, the predictive power of HE4 alone was limited. Recent single-cell RNA sequencing showed that seven genes were upregulated in both EAH and EEC, with DKK4, CST1, and NOTUM highly expressed only in EEC 31. These genes showed promise as biomarkers for detecting concurrent EC from EAH 31. Future research should integrate these genes with serum HE4 to develop a predictive model for better clinical accuracy and to avoid ineffective treatment and poor prognoses.

Currently, serum HE4 was most used with thresholds for detecting ovarian cancers. However, due to the distinct clinical and molecular characteristics of EC compared to ovarian cancers, no consensus on the appropriate HE4 thresholds for EC has been reached yet. Serum HE4 levels were influenced by multiple confounding factors, such as age, renal function, smoking habits, and other malignancies 9. The heterogeneity among study populations and the variability in testing techniques also presented challenges for comparing findings. In this study, we investigated the potential heterogeneity in population-related clinical characteristics and explored the correlation between serum HE4 levels and clinical factors. To predict the occurrence of concurrent EC, intermediate-high-risk EC, and non-candidates for fertility-preserving treatment, we determined different cut-offs for HE4 by using AUC analyses. It was observed that these HE4 levels demonstrated satisfactory predictive capability. However, our study lacked external validation, and further prospective multicenter studies were still needed to verify and determine the optimal cut-offs of HE4 levels in different predictive circumstances.

By using multivariate analysis, our results in preoperative-EAH patients indicated that these factors, such as age, BMI, and diabetes, did not serve as significant independent predictors of concurrent EC. This finding was consistent with recent research by Luca Giannella et al 32. Using predictive models including regressions and artificial neural networks, their results suggested that although BMI might be associated with EC, its predictive power was clinically inadequate when assessed on a validation set 32. Age and diabetes also led to similar results 32.

In our study of 715 preoperative-EAH patients, not all individuals underwent endometrial lesion sampling through HSC. Specifically, 42.7% (305/715) of patients underwent D&C with HSC, while 54.7% (391/715) and 2.7% (19/715) received D&C alone without HSC and Pipelle biopsy respectively (Table 1-2). Research by Luca Giannella et al. demonstrated that endoscopic endometrial sampling has a lower rate of underestimating EC compared to D&C (28% vs. 35%, respectively) 32. They identified hysteroscopic endometrial resection (HSC-res) as the most accurate method for endometrial sampling 33, with the ability to obtain substantial tissue samples under direct visualization and targeted sampling of significant lesions. This was followed in accuracy by hysteroscopy-guided biopsy, with D&C being the least precise 33. Consequently, they recommended HSC-res for women with EAH preoperatively to minimize the risk of undetected EC during hysterectomy 33. Despite in multivariate analysis, we did not find significant correlation between the endometrial sampling methods and final diagnoses, the hysteroscopy with D&C still showed the best detective efficacy on concurrent intermediate-high-risk EC in univariate analysis. In future perspective studies regarding detection of EC, hysteroscopy with D&C should be recommended as first option.

### Strengths and limitations

This study has several strengths. First, leveraging a large sample size, we were the first to evaluate the predictive value of serum HE4 in identifying concurrent intermediate- to high-risk EC among patients with preoperative EAH. We also determined optimal HE4 cut-off values under various predictive conditions, while controlling for potential confounders affecting HE4 levels. Additionally, all pathological specimens were reviewed by gynecologic pathologists at a single tertiary center, ensuring diagnostic consistency and high quality. Finally, we developed a clinically applicable nomogram for individualized risk prediction. However, several limitations should be acknowledged. First, the study was a single-center retrospective analysis. Second, only 42.7% of preoperative-EAH patients underwent HSC with D&C for endometrial sampling, while 54.7% and 2.7% received D&C without HSC and Pipelle biopsy, respectively. Although multivariate analysis showed no significant association between sampling method and final diagnosis, HSC with D&C demonstrated superior detection of concurrent intermediate- to high-risk EC in univariate analysis. Therefore, HSC with D&C should be considered the preferred sampling method in future prospective studies. Moreover, while the nomogram was internally validated, external validation is still needed. The model also included only clinicopathological parameters and serum biomarkers; future studies should incorporate additional biomarkers to enhance predictive performance and clinical utility, ideally within a prospective, multicenter study design.

## Conclusion

In conclusion, serum HE4 demonstrated good predictive capability for concurrent EC, intermediate-high-risk EC, and non-candidates for fertility-preserving treatment in preoperative-EAH patients. These findings highlighted its potential as a promising biomarker for diagnostic models and predicting the feasibility of fertility preservation. To predict intermediate-high-risk EC, we developed a nomogram based on serum HE4 levels and menopausal status, which indicated significant predictive accuracy and clinical applicability. This nomogram could assist in identifying those preoperative-EAH women at increased risk for concurrent intermediate-high-risk EC, improving decision-making and personalized management clinically. However, these findings should be further validated through multicenter prospective studies to determine the optimal cut-offs for serum HE4 levels. Additionally, more non-invasive biomarkers and imaging indicators should be explored and integrated into the predictive model for preoperative-EAH populations.

## Supplementary Material

Supplementary figures.

## Figures and Tables

**Table 1:** Clinical characteristics of all 715 patients diagnosed with EAH and EC by final histopathology.

Characteristics	Number	Total (n = 715)	Final-EAH (n = 528)	Final-EC (n = 187)	*P* value
Median (interquartile range)					
Age (years)	715	47 (42-51)	46.5 (42-50.75)	48 (43-55)	0.001
BMI (kg/m^2^)	674	24.58 (22.43-27.49)	24.58 (22.42-27.30)	24.58 (22.43-28.27)	0.583
HE4 (pmol/L)	715	47.70 (40.70-55.60)	45.94 (39.60-52.68)	52.70 (44.60-65.10)	< 0.001
CA125 (U/ml)	705	15.30 (11.54-21.74)	15.10 (11.28-21.27)	16.67 (11.86-23.59)	0.085
Endometrial thickness by ultrasound (mm)	623	8 (5-11)	7 (5-10)	9 (5-13)	0.013
Number (%)					
Age (years)	715				< 0.001
≤ 40		156 (21.8%)	120 (22.7%)	36 (19.3%)	
41-50		352 (49.2%)	276 (52.3%)	76 (40.6%)	
51-60		175 (24.5%)	118 (22.3%)	57 (30.5%)	
> 60		32 (4.5%)	14 (2.7%)	18 (9.6%)	
BMI (kg/m^2^)	674				0.243
Not obese (< 28)		521 (77.3%)	389 (78.4%)	132 (74.2%)	
Obese (≥ 28)		153 (22.7%)	107 (21.6%)	46 (25.8%)	
Menopausal status	715				< 0.001
Premenopausal		583 (81.5%)	459 (86.9%)	124 (66.3%)	
Postmenopausal time < 2 years		37 (5.2%)	23 (4.4%)	14 (7.5%)	
Postmenopausal time ≥ 2 - < 5 years		37 (5.2%)	21 (4.0%)	16 (8.6%)	
Postmenopausal time ≥ 5 years		58 (8.1%)	25 (4.7%)	33 (17.6%)	
Fertility	715				0.611
Parous		678 (94.8%)	502 (95.1%)	176 (94.1%)	
Nulliparous		37 (5.2%)	26 (4.9%)	11 (5.9%)	
Diabetes	703				0.022
NO		640 (91.0%)	481 (92.5%)	159 (86.9%)	
YES		63 (9.0%)	39 (7.5%)	24 (13.1%)	
Hypertension	656				0.007
NO		477 (72.7%)	364 (75.5%)	113 (64.9%)	
YES		179 (27.3%)	118 (24.5%)	61 (35.1%)	
FBG (mmol/L)	693				0.503
< 7.0		658 (94.9%)	485 (95.3%)	173 (94.0%)	
≥ 7.0		35 (5.1%)	24 (4.7%)	11 (6.0%)	
CA125 (U/ml)	705				0.249
< 35		629 (89.2%)	469 (90.0%)	160 (87.0%)	
≥ 35		76 (10.8%)	52 (10.0%)	24 (13.0%)	
eGFR (ml/min/1.73m^2^)	687				0.260
≥ 90		670 (97.5%)	496 (98.0%)	174 (96.1%)	
< 90		17 (2.5%)	10 (2.0%)	7 (3.9%)	
Sampling method	715				0.930
D&C alone		391 (54.7%)	291 (55.1%)	100 (53.5%)	
D&C with HSC		305 (42.7%)	223 (42.2%)	82 (43.9%)	
Pipelle biopsy		19 (2.7%)	14 (2.7%)	5 (2.7%)	
Endometrial thickness by ultrasound (mm)	623				< 0.001
< 20		609 (97.8%)	464 (99.6%)	145 (92.4%)	
≥ 20		14 (2.2%)	2 (0.4%)	12 (7.6%)	

Notes: Data were presented as median (interquartile range) or number (%). Percentage calculations excluded missing data. Missing data included 41 cases for BMI, 10 for CA125, 92 for endometrial thickness by ultrasound, 12 for diabetes, 59 for hypertension, 22 for FBG, and 28 for eGFR value. *P* value: difference between the final-EAH group and final-EC group. Significant *P* value < 0.05. Abbreviations: EAH, endometrial atypical hyperplasia; EC, endometrial cancer; final-EAH, endometrial atypical hyperplasia diagnosed by final histopathology; final-EC, endometrial cancer diagnosed by final histopathology; BMI, body mass index; HE4, human epididymis protein 4; CA125, cancer antigen 125; FBG, fasting blood glucose; eGFR, estimated glomerular filtration rate; D&C, dilatation and curettage; HSC, hysteroscopy.

**Table 2:** Clinical and Pathological factors in relation to serum levels of HE4 in all 715 preoperative-EAH patients or in 187 final-EC patients.

Variables	Number	Patients	HE4 (pmol/L)	Spearman's Rho	Spearman's *P* value
			Median (interquartile range)		
In 715 preoperative-EAH patients					
All patients	715	715	47.70 (40.70-55.60)	-	NS
Age (years)	715			0.232	< 0.001
≤ 40		156 (21.8%)	44.25 (38.30-51.55)		
41-50		352 (49.2%)	47.35 (40.45-53.60)		
51-60		175 (24.5%)	50.00 (41.95-59.30)		
> 60		32 (4.5%)	60.00 (51.15-81.35)		
BMI (kg/m^2^)	674			-0.028	0.475
Not obese (< 28)		521 (77.3%)	47.60 (41.10-55.60)		
Obese (≥ 28)		153 (22.7%)	46.60 (38.60-55.10)		
Menopausal status	715			0.157	< 0.001
Premenopausal		583 (81.5%)	47.00 (40.10-54.15)		
Postmenopausal time < 2 years		37 (5.2%)	45.20 (40.30-54.20)		
Postmenopausal time ≥ 2 - < 5 years		37 (5.2%)	47.70 (41.90-55.60)		
Postmenopausal time ≥ 5 years		58 (8.1%)	58.00 (49.50-71.60)		
Fertility	715			-0.005	0.887
Parous		678 (94.8%)	47.70 (40.70-55.60)		
Nulliparous		37 (5.2%)	46.70 (40.95-54.10)		
Diabetes	703			-0.033	0.383
NO		640 (91.0%)	47.70 (40.80-55.65)		
YES		63 (9.0%)	44.40 (38.90-54.50)		
Hypertension	656			0.118	0.003
NO		477 (72.7%)	47.30 (40.60-55.00)		
YES		179 (27.3%)	50.20 (42.50-60.15)		
CA125 (U/ml)	705			0.151	< 0.001
< 35		629 (89.2%)	47.10 (40.30-54.90)		
≥ 35		76 (10.8%)	52.05 (45.40-68.05)		
Sampling method	715			-0.059	0.113
D&C alone		391 (54.7%)	48.30 (41.15-56.90)		
D&C with HSC		305 (42.7%)	46.40 (40.30-53.20)		
Pipelle biopsy		19 (2.7%)	51.40 (44.45-60.70)		
Endometrial thickness by ultrasound (mm)	623			0.179	< 0.001
< 20		609 (97.8%)	46.80 (40.30-54.25)		
≥ 20		14 (2.2%)	97.35 (53.48-136.63)		
In 187 final-EC patients					
Histology	187			-0.024	0.744
Endometrioid		184 (98.4%)	52.75 (44.75-64.95)		
Others		3 (1.6%)	45.2 (40.40-113.80)		
FIGO stage (2009)	187			0.109	0.139
I		175 (93.6%)	52.10 (44.40-63.90)		
II		5 (2.7%)	72.70 (51.65-76.80)		
III		7 (3.7%)	54.20 (47.80-76.10)		
IV		0	-		
Grade	182			0.056	0.450
1		173 (95.6%)	52.10 (44.45-63.30)		
2		7 (3.9%)	71.40 (49.70-76.20)		
3		1 (0.6%)	46.70		
Myometrial invasion	187			0.253	< 0.001
No or < 50%		171 (91.4%)	52.00 (44.40-61.50)		
≥ 50%		16 (8.6%)	76.50 (57.50-83.95)		
Cervical interstitial infiltration	187			0.105	0.151
(-)		182 (97.3%)	52.40 (44.50-63.90)		
(+)		5 (2.7%)	72.70 (52.00-76.20)		
Lymphovascular space invasion	105			0.126	0.201
(-)		92 (87.6%)	54.65 (45.65-70.85)		
(+)		13 (12.4%)	76.10 (54.90-84.90)		
Pelvic lymph node	19			-0.094	0.702
(-)		17 (89.5%)	59.90 (52.00-77.40)		
(+)		2 (10.5%)	61.95 (47.80-76.10)		
Para-aortic lymph node	11			-0.224	0.509
(-)		9 (81.8%)	60.40 (53.20-84.20)		
(+)		2 (18.2%)	61.95 (47.80-76.10)		
MILF	187			0.164	0.025
(-)		179 (95.7%)	52.00 (44.45-63.30)		
(+)		8 (4.3%)	76.50 (55.00-81.15)		
Pathological maximum diameter (cm)	138			0.187	0.028
< 2.0		37 (26.8%)	49.10 (41.40-59.30)		
≥ 2.0		101 (73.2%)	55.10 (45.55-68.10)		
Risk stratification (based on NCCN 2024)	187			0.258	< 0.001
Low-risk EC		144 (77.01%)	51.25 (43.75-60.75)		
Intermediate-high-risk EC		43 (22.99%)	60.10 (51.65-78.10)		

Notes: Data were presented as median (interquartile range) or number (%). Significant *P* value < 0.05. Low-risk EC was defined as: (a) endometrioid endometrial cancer, stage IA (FIGO2009), grade 1-2, without lymph-vascular space invasion, age < 60 years; (b) endometrioid endometrial cancer, stage IA (FIGO2009), grade 3, without lymph-vascular space invasion, age < 60 years, without myometrial invasion. All other EC cases except for low-risk EC were defined as intermediate-high-risk EC, based on the NCCN guidelines (version 3.2024). Abbreviations: HE4, human epididymis protein 4; EAH, endometrial atypical hyperplasia; BMI, body mass index; CA125, cancer antigen 125; D&C, dilatation and curettage; HSC, hysteroscopy; final-EC, endometrial cancer diagnosed by final histopathology; FIGO, the International Federation of Gynecology and Obstetrics; MELF, microcystic, elongated, and fragmented; NCCN, National Comprehensive Cancer Network.

## Abbreviations

AIC: Akaike Information Criterion
AUC: area under the curve
BIC: Bayesian Information Criterion
BMI: body mass index
CA125: cancer antigen 125
CI: confidence interval
C-index: concordance index
D&C: dilatation and curettage
DCA: decision curve analysis
EAH: endometrial atypical hyperplasia
EC: endometrial cancer
EEC: early-stage endometrial cancer
eGFR: estimated glomerular filtration rate
EPV: events per variable
FBG: fasting blood glucose
FIGO: the International Federation of Gynecology and Obstetrics
final-EAH: endometrial atypical hyperplasia diagnosed by final histopathology
final-EC: endometrial cancer diagnosed by final histopathology
FPT: fertility-preserving therapy
HE4: human epididymis protein 4
HSC: hysteroscopy
HSC-res: hysteroscopic endometrial resection
NCCN: National Comprehensive Cancer Network
OR: odds ratio
preoperative-EAH: endometrial atypical hyperplasia before definitive surgery
ROC: receiver operator characteristic
Scr: serum creatinine
SLN: sentinel lymph node
WHO: the World Health Organization
MELF: microcystic, elongated, and fragmented
