# Supplementary material for: Human Epididymis Protein 4 Predicted Concurrent Intermediate-high-risk Endometrial Cancer and Eligibility of Fertility-sparing Treatment for Patients Diagnosed with Endometrial Atypical Hyperplasia Before Surgery
Source: Int J Med Sci. 2025 Jul 11;22(13):3292–303. doi: 10.7150/ijms.115170 (PMC12320788; doi:10.7150/ijms.115170)

Supplementary Materials

Figure S1. The flowchart of patient selection.

Abbreviations: EAH, endometrial atypical hyperplasia; HE4, human epididymis protein 4.

Figure S2. The ROC curves of serum levels of HE4 in different predicting models. (A-B) The value of HE4 in predicting concurrent EC and intermediate-high-risk EC in all 715 preoperative-EAH patients, based on 2024 NCCN guidelines. (C) The value of HE4 in predicting non-candidates of fertility-sparing treatment in 285 young preoperative-EAH patients.

Notes: Low-risk EC was defined as: (a) endometrioid endometrial cancer, stage IA (FIGO2009), grade 1-2, without lymph-vascular space invasion, age < 60 years; (b) endometrioid endometrial cancer, stage IA (FIGO2009), grade 3, without lymph-vascular space invasion, age < 60 years, without myometrial invasion. All other EC cases except for low-risk EC were defined as intermediate-high-risk EC, based on the NCCN guidelines (version 3.2024).

Abbreviations: HE4, human epididymis protein 4; EAH, endometrial atypical hyperplasia; EC, endometrial cancer; final-EC, endometrial cancer diagnosed by final histopathology; AUC, area under the curve; ROC, receiver operator characteristic.

Figure S3. Univariate and multivariate analyses of factors predicting final-EC for 715 preoperative-EAH patients with and without endometrial thickness by ultrasound.

Notes: Missing data included 41 cases for BMI, 12 for diabetes, 59 for hypertension, 22 for FBG, 10 for CA125, and 92 for endometrial thickness by ultrasound. The categorical variable of serum HE4 levels had a cut-off as 43.5 pmol/L. In multivariate logistic analysis with available endometrial thickness by ultrasound, OR adjusted for age, BMI, menopausal status, diabetes, hypertension, HE4 value, CA125 value, and endometrial thickness by ultrasound. In multivariate logistic analysis without available endometrial thickness by ultrasound, OR adjusted for age, BMI, menopausal status, diabetes, hypertension, HE4 value, and CA125 value. Significant P value < 0.05.

Abbreviations: EAH, endometrial atypical hyperplasia; EC, endometrial cancer; BMI, body mass index; FBG, fasting blood glucose; HE4, human epididymis protein 4; CA125, cancer antigen 125; D&C, dilatation and curettage; HSC, hysteroscopy; OR, odds ratio; CI, confidence interval.

Figure S4. Univariate and multivariate analyses of factors predicting concurrent intermediate-high-risk EC for 715 preoperative-EAH patients incorporating hypertension.

Notes: Missing data included 41 cases for BMI, 12 for diabetes, 59 for hypertension, 22 for FBG, 10 for CA125, and 92 for endometrial thickness by ultrasound. The categorical variable of serum HE4 levels had a cut-off as 53.15 pmol/L. In multivariate logistic analysis, OR adjusted for age, menopausal status, hypertension, HE4 value, and CA125 value. Significant *P* value < 0.05. Low-risk EC was defined as: (a) endometrioid endometrial cancer, stage IA (FIGO2009), grade 1-2, without lymph-vascular space invasion, age < 60 years; (b) endometrioid endometrial cancer, stage IA (FIGO2009), grade 3, without lymph-vascular space invasion, age < 60 years, without myometrial invasion. All other EC cases except for low-risk EC were defined as intermediate-high-risk EC, based on the NCCN guidelines (version 3.2024).

Abbreviations: EAH, endometrial atypical hyperplasia; EC, endometrial cancer; BMI, body mass index; FBG, fasting blood glucose; HE4, human epididymis protein 4; CA125, cancer antigen 125; OR, odds ratio; CI, confidence interval; D&C, dilatation and curettage; HSC, hysteroscopy.

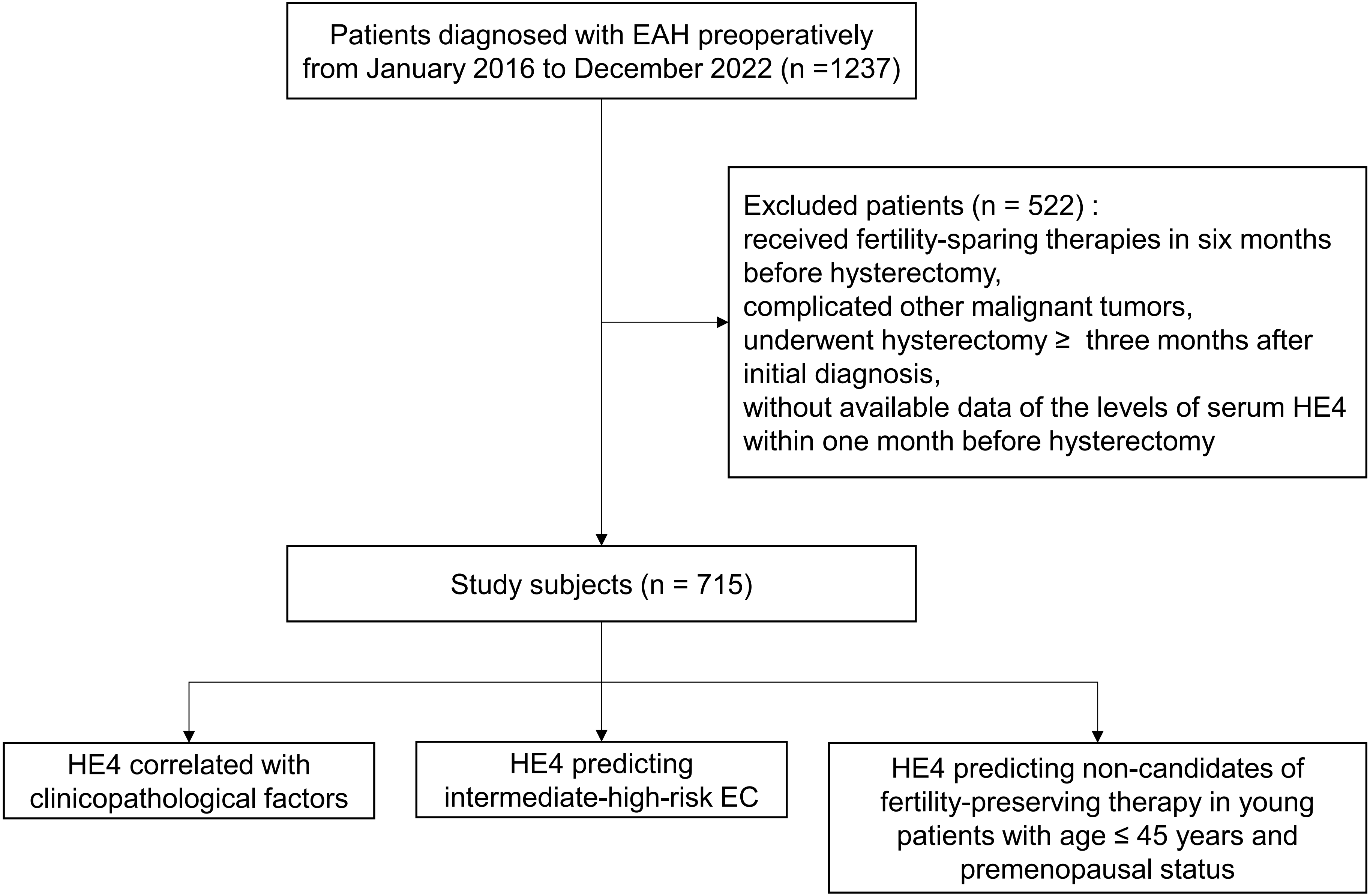
Patients diagnosed with EAH preoperatively from January 2016 to December 2022 (n =1237)
Excluded patients (n = 522) :
received fertility-sparing therapies in six months before hysterectomy,
complicated other malignant tumors,
underwent hysterectomy ≥ three months after initial diagnosis,
without available data of the levels of serum HE4 within one month before hysterectomy
Study subjects (n = 715)
HE4 correlated with clinicopathological factors
HE4 predicting intermediate-high-risk EC
HE4 predicting non-candidates of fertility-preserving therapy in young patients with age ≤ 45 years and premenopausal status

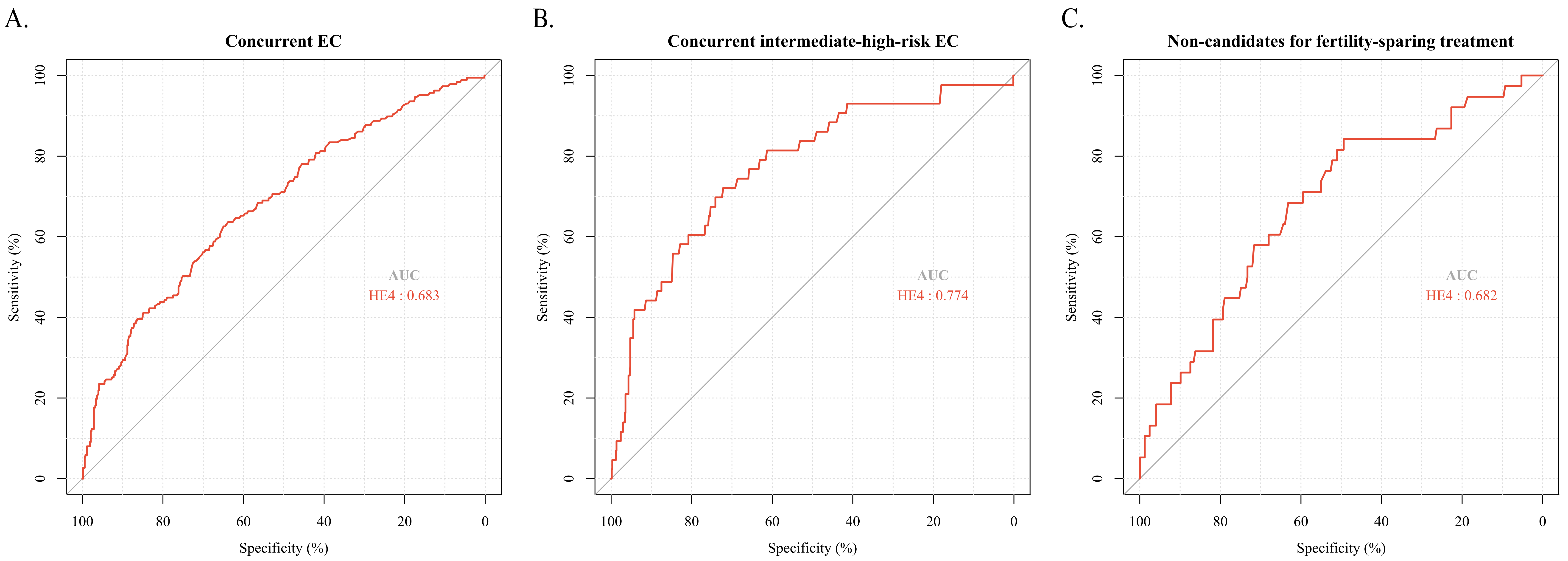

A.
Concurrent EC
AUC
HE4 : 0.683
B.
Concurrent intermediate-high-risk EC
AUC
HE4 : 0.774
C.
Non-candidates for fertility-sparing treatment
AUC
HE4 : 0.682
Sensitivity (%)
Specificity (%)
100
80
60
40
20
0

Univariate Analysis

Variables	Total No.	OR (95% CI)	*P*
**Age (years):**	715		
< 60	677 (94.7%)	Reference	
≥ 60	38 (5.3%)	1.96 (3.80-7.38)	< 0.001
**BMI (kg/m²):**	674		
< 28	521 (77.3%)	Reference	
≥ 28	153 (22.7%)	1.27 (0.85-1.89)	0.244
**Menopausal status:**	715		
Premenopausal	583 (81.5%)	Reference	
Postmenopausal time < 2 years	37 (5.2%)	2.25 (1.13-4.51)	0.022
Postmenopausal time ≥ 2 - < 5 years	37 (5.2%)	2.82 (1.43-5.57)	0.003
Postmenopausal time ≥ 5 years	58 (8.1%)	4.89 (2.80-8.52)	< 0.001
**Fertility**	715		
Parous	678 (94.8%)	Reference	
Nulliparous	37 (5.2%)	1.21 (0.58-2.49)	0.612
**Diabetes:**	703		
No	640 (91.0%)	Reference	
Yes	63 (9.0%)	1.86 (1.09-3.19)	0.024
**Hypertension:**	656		
No	477 (72.7%)	Reference	
Yes	179 (27.3%)	1.67 (1.15-2.42)	0.008
**FBG (mmol/L):**	693		
< 7.0	658 (94.9%)	Reference	
≥ 7.0	35 (5.1%)	1.29 (0.62-2.68)	0.503
**HE4 (pmol/L):**	715		
< 43.5	258 (36.1%)	Reference	
≥ 43.5	457 (63.9%)	3.04 (2.04-4.55)	< 0.001
**CA125 (U/mL):**	705		
< 35	629 (89.2%)	Reference	
≥ 35	76 (10.8%)	1.35 (0.81-2.27)	0.251
**Sampling method:**	715		
D&C alone	391 (54.7%)	Reference	
D&C with HSC	305 (42.7%)	1.07 (0.76-1.50)	0.696
Pipelle biopsy	19 (2.7%)	1.04 (0.37-2.96)	0.942
**Endometrial thickness by ultrasound (mm)**	623		
< 20	609 (97.8%)	Reference	
≥ 20	14 (2.2%)	19.20 (4.25-86.78)	< 0.001

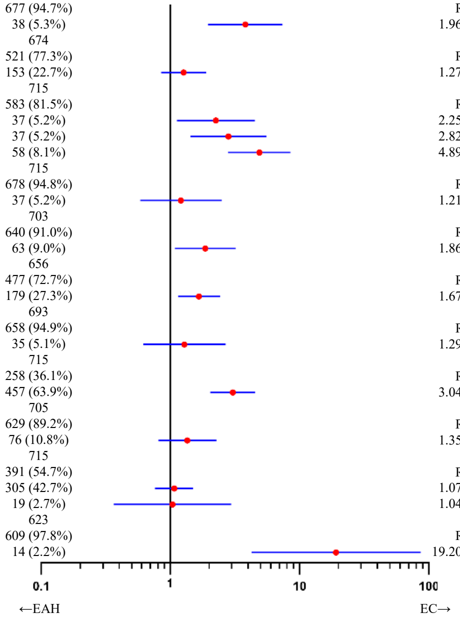

Multivariate Analysis with Available Endometrial Thickness

Variables	Adjusted OR (95% CI)	*P*
**Age (years):**		
< 60	Reference	
≥ 60	0.30 (0.09-1.04)	0.057
**BMI (kg/m²):**		
< 28	Reference	
≥ 28	1.09 (0.65-1.86)	0.740
**Menopausal status:**		
Premenopausal	Reference	
Postmenopausal time < 2 years	2.64 (1.07-6.52)	0.036
Postmenopausal time ≥ 2 - < 5 years	4.09 (1.71-9.81)	0.002
Postmenopausal time ≥ 5 years	7.09 (2.52-19.98)	< 0.001
**Diabetes:**		
No	Reference	
Yes	1.50 (0.71-3.19)	0.291
**Hypertension:**		
No	Reference	
Yes	0.94 (0.56-1.56)	0.798
**HE4 (pmol/L):**		
< 43.5	Reference	
≥ 43.5	2.79 (1.72-4.54)	< 0.001
**CA125 (U/mL):**		
< 35	Reference	
≥ 35	1.16 (0.59-2.27)	0.675
**Endometrial thickness by ultrasound (mm)**		
< 20	Reference	
≥ 20	29.35 (3.60-239.31)	0.002

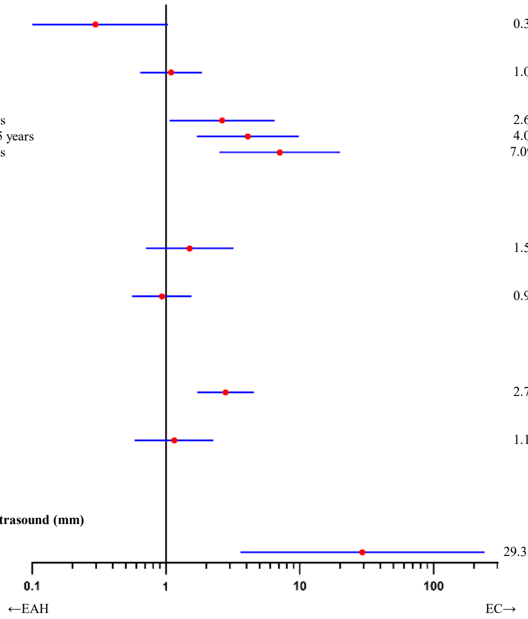

Multivariate Analysis without Available Endometrial Thickness

Variables	Adjusted OR (95% CI)	*P*
**Age (years):**		
< 60	Reference	
≥ 60	0.44 (0.15-1.31)	0.140
**BMI (kg/m²):**		
< 28	Reference	
≥ 28	1.12 (0.70-1.80)	0.642
**Menopausal status:**		
Premenopausal	Reference	
Postmenopausal time < 2 years	2.44 (1.08-5.53)	0.032
Postmenopausal time ≥ 2 - < 5 years	3.76 (1.71-8.28)	0.001
Postmenopausal time ≥ 5 years	5.76 (2.30-14.42)	< 0.001
**Diabetes:**		
No	Reference	
Yes	1.60 (0.82-3.12)	0.170
**Hypertension:**		
No	Reference	
Yes	1.11 (0.70-1.74)	0.669
**HE4 (pmol/L):**		
< 43.5	Reference	
≥ 43.5	2.61 (1.68-4.08)	< 0.001
**CA125 (U/mL):**		
< 35	Reference	
≥ 35	1.27 (0.70-2.27)	0.431

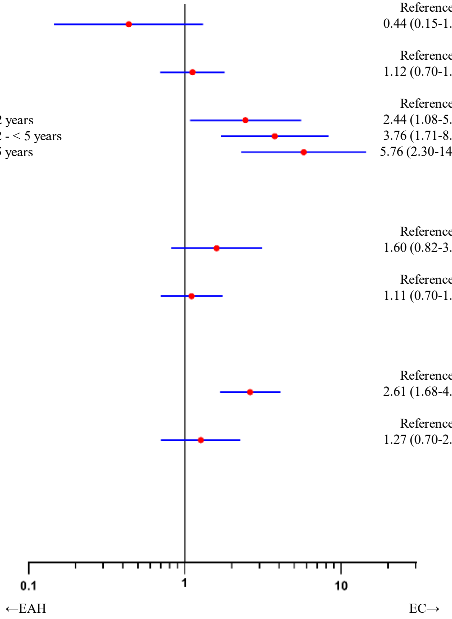

Univariate Analysis

Variables	Total No.	OR (95% CI)	*P*
**BMI (kg/m²):**	674		
< 28	521 (77.3%)	Reference	
≥ 28	153 (22.7%)	1.36 (0.66-2.81)	0.399
**Menopausal status:**	715		
Premenopausal	583 (81.5%)	Reference	
Postmenopausal time < 5 years	74 (10.3%)	2.95 (1.03-8.43)	0.044
Postmenopausal time ≥ 5 years	58 (8.1%)	28.69 (13.63-60.40)	< 0.001
**Fertility:**	715		
Parous	678 (94.8%)	Reference	
Nulliparous	37 (5.2%)	0.89 (0.21-3.82)	0.873
**Diabetes:**	703		
No	640 (91.0%)	Reference	
Yes	63 (9.0%)	2.16 (0.92-5.09)	0.078
**Hypertension:**	656		
No	477 (72.7%)	Reference	
Yes	179 (27.3%)	2.43 (1.26-4.67)	0.008
**FBG (mmol/L):**	693		
< 7.0	658 (94.9%)	Reference	
≥ 7.0	35 (5.1%)	2.11 (0.71-6.27)	0.181
**HE4 (pmol/L):**	715		
< 53.15	497 (69.5%)	Reference	
≥ 53.15	218 (30.5%)	6.70 (3.37-13.32)	< 0.001
**CA125 (U/mL):**	705		
< 35	629 (89.2%)	Reference	
≥ 35	76 (10.8%)	2.74 (1.29-5.80)	0.009
**Sampling method:**	715		
D&C alone	391 (54.7%)	Reference	
D&C with HSC	305 (42.7%)	2.34 (1.22-4.49)	0.011
Pipelle biopsy	19 (2.7%)	2.95 (0.62-13.94)	0.172
**Endometrial thickness by ultrasound (mm)**	623		
< 20	609 (97.8%)	Reference	
≥ 20	14 (2.2%)	1.43 (0.18-11.32)	0.732

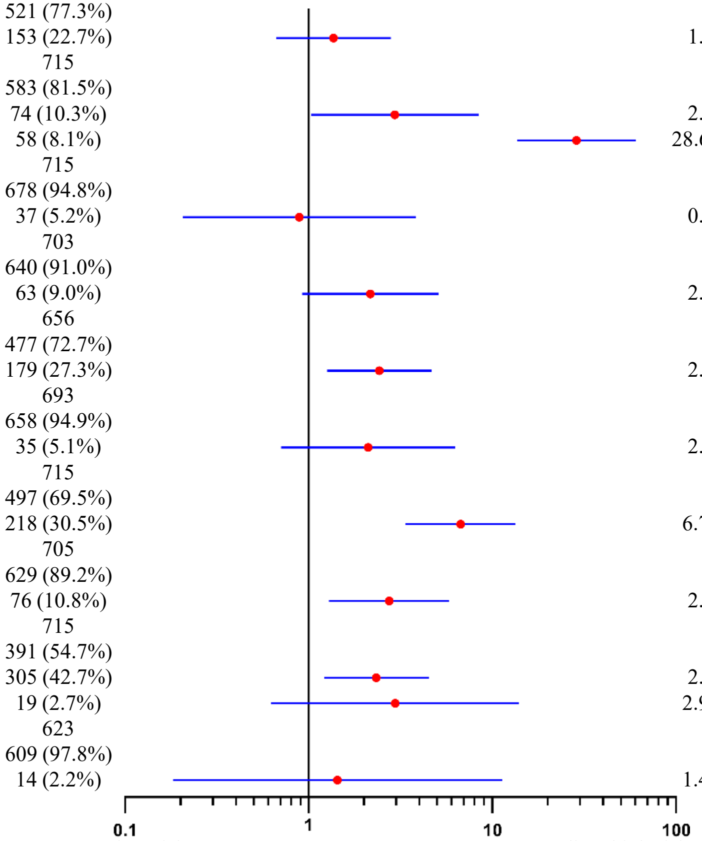

Multivariate Analysis

Variables	Adjusted OR (95% CI)	*P*
**Menopausal status:**		
Premenopausal	Reference	
Postmenopausal time < 5 years	3.15 (1.04-9.51)	0.042
Postmenopausal time ≥ 5 years	16.22 (6.87-38.31)	< 0.001
**Hypertension:**		
No	Reference	
Yes	1.35 (0.61-2.99)	0. 454
**HE4 (pmol/L):**		
< 53.15	Reference	
≥ 53.15	3.49 (1.55-7.83)	0.002
**CA125 (U/mL):**		
< 35	Reference	
≥ 35	2.11 (0.77-5.76)	0.146
**Sampling method:**		
D&C alone	Reference	
D&C with HSC	1.62 (0.75-3.53)	0.221
Pipelle biopsy	1.55 (0.25-9.70)	0.638

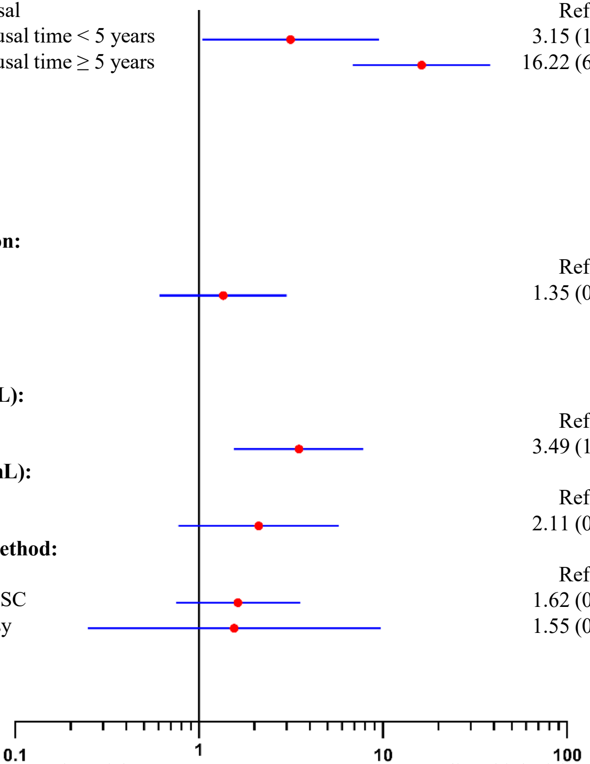

Supplement: Supplementary file 1 — Supplementary figures. [file ijmsv22p3292s1.pdf]
